# Natural Killer T Cells in Liver Ischemia–Reperfusion Injury

**DOI:** 10.3390/jcm6040041

**Published:** 2017-04-01

**Authors:** Michael A. Zimmerman, Alicia Martin, Jennifer Yee, Jennifer Schiller, Johnny C. Hong

**Affiliations:** 1 Department of Surgery, Division of Transplant Surgery, Medical College of Wisconsin, Milwaukee, WI 53226, USA; amartin@mcw.edu (A.M.); jyee@mcw.edu (J.Y.); jennie.schiller@bcw.edu (J.S.); jhong@mcw.edu (J.C.H.); 2 Histocompatibility and Immunogenetics, Blood Center of Wisconsin, Milwaukee, WI 53201, USA

**Keywords:** lymphocyte, liver, ischemia, reperfusion, transplant

## Abstract

Restoration of blood flow to an ischemic organ results in significant tissue injury. In the field of liver transplantation, ischemia–reperfusion injury (IRI) has proven to be a formidable clinical obstacle. In addition to metabolic stress and inflammation, IRI results in profound graft dysfunction and loss. The severity of IRI further limits the ability to expand the donor pool by using partial grafts and marginal organs. As such, the inflammatory response to reperfusion of the liver continues to be an area of intense investigation. Among the various leukocytes involved in IRI, new insights suggest that natural killer T (NKT) cells may be a central driver of hepatocellular injury. Herein, we examine recent experimental observations that provide a mechanistic link between NKT cell recruitment to liver and post-perfusion tissue injury.

## 1. Introduction

Acute cessation of blood flow to an end organ results in severe localized hypoxia [1]. Restoration of perfusion results in significant cellular injury and subsequent organ dysfunction termed ischemia–reperfusion injury (IRI). IRI of the liver is frequently encountered following hemorrhagic shock, surgical resection, and solid organ transplant. In the setting of liver transplantation, two defined periods of ischemia are present prior to reperfusion [2]. Initially, “cold” ischemia promotes sinusoidal endothelial cell injury leading to microcirculatory disruption. Cellular injury occurs during the organ procurement and cold storage phases, which are variable in length. This is followed by “warm” ischemia, the time during which the liver is sewn into the recipient. While the warm and cold ischemic phases are characteristically different, they may share common mechanisms of tissue injury leading to graft dysfunction. Unfortunately, both phases are obligatory in liver transplant. Clinically, attenuating cell death and dysfunction will improve long-term outcomes in the recipient. In addition, reducing the severity of “reperfusion syndrome” will facilitate the use of marginal grafts, thus expanding the donor pool.

Prolonged oxygen deprivation results in ATP depletion, cellular conversion to anaerobic metabolism, and ultimately cell death. While reestablishing blood flow is required to restore cellular function, this results in a profound inflammatory response. Pathologically, tissue injury is characterized by endothelial injury, microvascular disruption, increased apoptosis, and ultimately necrosis [3]. Preclinical studies over the last two decades suggest that liver IRI in both phases is characterized by local inflammation mediated by both innate and adaptive immune responses. This includes Kupffer cell activation, neutrophil production of cytokines and chemokines, as well as lymphocyte/monocyte infiltration [2,4].

With a robust inflammatory cascade dominated by the innate response initially, adaptive responses appear to be dependent on CD4^+^ T cells. This may be particularly important in multiple phases of ischemic injury. Zhai and colleagues note that crosstalk between innate and adaptive responses triggered by IRI converts the liver into an inflammatory organ in a sterile environment [4]. Following reperfusion, CD4^+^ T lymphocytes are activated and recruited to the liver. Depending on the subtype, these lymphocytes may contribute to, or directly inhibit, hepatocellular injury. Despite accumulating evidence defining the role of innate and adaptive responses in hepatic IRI, the specific role of various T cell subsets continues to be defined.

## 2. T Lymphocytes and Post-Ischemic Liver Pathology

Experimental observations made over the last three decades suggest that neutrophils play a major role in tissue injury in the early phase following reestablishment of blood flow [5]. This response is biphasic with early cellular damage within 6 h of reperfusion and robust neutrophil infiltration at 24 h [6,7,8,9]. Several studies reported by Jaescheke and colleagues in the early 1990s clearly demonstrated that antibody-mediated neutrophil depletion attenuates IRI injury in rodent models [10,11]. While these observations have been confirmed by numerous investigators, the precise role of T lymphocytes in IRI continues to be defined and may be linked to a specific subtype. As the adult liver ages, it harbors roughly 10^10^ resident lymphocytes [12,13]. These cells alter inflammatory responses via paracrine secretion of either pro- or anti-inflammatory mediators. Production of these soluble mediators can directly alter the phenotype of other resident lymphocytes as well as stimulate other inflammatory cells in the liver [14]. Despite these general influences, it remains unclear exactly when and how T lymphocytes directly promote tissue injury in the ischemic liver upon reperfusion.

In 1997, Zwacka and colleagues made several critical observations pertaining to the role of T lymphocytes in liver IRI (Table 1). Using a murine model of warm ischemia, they observed that T-lymphocytes are key mediators of subacute neutrophil inflammatory responses in liver IRI [15]. Employing partial liver ischemia, they compared reperfusion injury between wild type and athymic mice. While they noted similar levels of acute injury (3–6 h post-reperfusion), the subacute phase (16–20 h) was dramatically different. Serum transaminases and degree of histologic necrosis was significantly reduced in the T cell-deficient mice. This was associated with a 10-fold reduction in neutrophil infiltration. Similarly, antibody depletion of CD4^+^ T lymphocytes in wild type mice resulted in a reduction of subacute injury and inflammation. Depletion of CD8^+^ T lymphocytes had no effect on transaminase levels or tissue injury. The authors concluded that these data suggest that CD4^+^ T lymphocytes are required for complete activation of post-reperfusion inflammatory responses and may be key regulators of neutrophil recruitment and amplification in the injured liver during the subacute phase.

As partial warm ischemia has become a widespread model employed in rodents, several groups have attempted to further define the role of T lymphocytes in acute and subacute phases of injury. Contrary to Zwacka, investigators at the University of California, Los Angeles, found a dramatic reduction in serum alanine aminotransferase (ALT) at just 4 h reperfusion in athymic mice (nu/nu) [16]. In this model, blockade of T lymphocyte signaling via CD154 resulted in a reduction of neutrophil accumulation, thus strengthening the argument that T lymphocyte signaling pathways (i.e., via CD154) are essential to liver injury as well as orchestrating neutrophil accumulation.

In addition to resident leukocytes, peripheral T lymphocytes are recruited to the liver as early as 60 min following IRI [17]. These recruited cells remained sequestered in the liver for at least 4 h post-reperfusion. Contrary to previous observations, mice that lack CD4^+^ T cells demonstrated a more robust biochemical injury at 8 h of reperfusion (ALT) than wild-type mice after 90 of initial ischemia. These findings were paradoxically associated with a reduction in neutrophil accumulation at the same time point. In addition, histologic sections demonstrate widespread areas of hemorrhagic necrosis and parenchymal cell destruction, with little evidence of neutrophil infiltration. Khandoga et al. demonstrated via videofluorescence microscopy, that CD4^+^ T cells accumulate and migrate in a transendothelial fashion into the sinusoids early following reperfusion [18]. Approximately 30% of CD4^+^ T cells were colocalized with platelets. CD4 deficiency disrupted this colocalization as well as reduced neutrophil transmigration and transaminase levels. Again, ALT levels were lower in CD4 deficient mice compared to wild type early post-reperfusion. Cumulatively, these data consistently characterize the early role of neutrophil-mediated hepatocellular injury in the setting of ischemia and subsequent reperfusion at various time points. Unfortunately, a mechanistic link between CD4^+^ lymphocyte recruitment/activation and neutrophil accumulation remains unclear [19]. Finally, the molecular tool employed by lymphocytes to directly inflict hepatocellular injury beyond the subacute phase in warm ischemia continues to be defined [20].

## 3. NKT Cells and Liver Pathology

Termed innate-like, natural killer T (NKT) cells appear to bridge the gap between innate and adaptive immune responses in numerous organs [21]. For example, NKT cell depletion or deficiency in mice significantly reduced IRI and decreased IFNγ-producing neutrophils in the kidney [22]. Similarly, lung dysfunction, injury, IL-17 expression, and neutrophil infiltration were attenuated following IRI in iNKT cell-deficient mice [23]. At present, there are at least two defined subsets (Type I and II) which may have opposing roles in liver. In general, NKT cells play an active role in autoimmunity, tumor immunosurveillance, diabetes, lupus, and a variety of liver diseases. For example, activation of NKT cells may be a critical factor in the acceleration of liver injury in the murine model of primary biliary cirrhosis [24,25,26]. Subsequent work suggests that the progression from subclinical to clinical disease may depend on natural ligand activation of NKT cells [27]. Mice deficient in NKT cells are protected from chemical induced hepatic fibrosis [28]. Similarly, data from rodent models and human tissues suggest NKT cells are critical drivers of fibrogenesis in non-alcoholic steatohepatitis [29].

These innate-like cells are abundant in the liver sinusoids and express conventional T cell receptors as well as surface receptors of NK cells (NK1.1 [mouse] and CD161/CD56 [human]) [30]. In mice, NKT cells make up approximately 30% of intrahepatic lymphocytes compared to nearly 10% in humans [31,32,33]. In contrast to traditional CD4^+^ T lymphocytes, NKT cells can be activated by antigen presenting cells (i.e., dendritic cells, macrophages, B cells, and thymocytes) that express CD1d. Importantly, CD1d is expressed by most cells in the liver. Interestingly, these cells constitutively express mRNA, encoding several proinflammatory cytokines (i.e., tumor necrosis factor-α, interferon-γ, Interleukin-6 and 4) [34], which are rapidly secreted on stimulation [35,36]. Rapid cytokine and chemokine production results in the accumulation of neutrophils and macrophages in the liver.

## 4. NKT Cell Activation and Liver IRI

Several investigators have recently focused on the role of NKT cells in liver IRI for three reasons: (1) the rapid development of liver IRI is not consistent with the time frame required for conventional T cell responses; (2) the majority of CD4^+^ T cells recruited to the liver early in the post-perfusion period consist mostly of NKT cells; and (3) NKT cells secrete cytokines rapidly upon stimulation. Early observations by Shimamura at Nigata University in Japan reported that the proportion of NKT cells rapidly expands in the liver following selective portal vein clamping and reperfusion injury [37]. While the proportion and number of NKT cells peaked in the liver at 10–20 h of reperfusion, the magnitude of liver injury is decreased by 50% in NKT cell-deficient mice as early as 6 h post-perfusion. Interestingly, NKT cells produce interferon-γ as early as 2 h following reperfusion [38]. As it is not surprising that antibody-mediated NKT depletion reduces biochemical and histological tissue injury in this model, adoptive transfer of NKT cells into lymphocyte-deficient mice restores injury to the level of wild type animals. A subset of experiments revealed a 58% reduction in serum ALT in mice treated with a synthetic adenosine 2A receptor (A_2A_R) agonist. Mechanistically, the A_2A_R agonist directly decreases IFN-γ production by activated NKT cells. A previous report from this group noted a dramatic reduction in IRI and proinflammatory cytokines/chemokine transcript levels following systemic treatment with an A_2A_R agonist [39]. These observations clearly suggest an anti-inflammatory role for purinergic signaling in warm hepatic ischemia.

Several investigators have shown that CD4^+^ T cells mediate neutrophil recruitment in the setting of hepatic reperfusion injury [17,18,38]. However, the mechanisms that are involved in direct tissue injury following restoration of hepatic blood flow are unclear. Using a partial warm ischemia model, Kuboki and colleagues found that NKT cells, but not NK cells, contribute to hepatic IRI by a CD1d-dependent activation of their T cell receptors [40]. Conversely, antibody depletion of NKT cells, or both NKT and NK cells significantly reduced liver injury following 8 h of reperfusion. Depletion of regulatory T cells had no effect on IRI. The authors speculate that although the precise mechanism of tissue injury is not defined in this study, the release of IFN-γ via recruited NKT cells might stimulate other proinflammatory cytokines which may augment liver injury and influence neutrophil function.

Exposure of the liver to ischemic injury in “sublethal” doses activates endogenous inflammatory pathways and/or protective mechanisms, rendering hepatocytes partially resistant to subsequent large scale ischemic insults [41,42,43,44]. Cao and colleagues from the University of Pittsburgh reported an interesting set of observations in a murine model of partial warm ischemia (portal vein occlusion for 60 min and 6 h of reperfusion) [45]. The authors employed a targeted strategy of biochemical NKT activation prior to induction of ischemia in an attempt to “precondition” the liver and augment tolerance to IRI. Preactivation of NKT cells was achieved by intraperitoneal injection of the glycolipid antigen α-galactosylceramide (α-GalCer) 60 min prior to hepatic ischemia. Preactivation resulted in a significant reduction in serum hepatic enzyme levels. The authors suggest that these effects may be mediated by interleukin-13 and adenosine receptor expression.

## 5. A Protective Role for Type II NKT Cells in the Liver

NKT cell subsets are more heterogeneous than originally thought [46]. NKT cells have been broadly divided into two distinct subtypes: Type I (invariant, iNKT) and Type II. Type II cells express a much more diverse T cell receptor than the iNKT cells [35,47] and lack reactivity to α-GalCer [48]. Contrary to activated Type I NKT cells, Type II cells have an anti-inflammatory influence, limiting tissue injury [49]. Sulfatide-mediated activation of Type II NKT cells is exclusive in that it does not activate B, T, or NK cells.

More abundant in humans than in mice, Type II NKT cells have a range of effects in various experimental models. For example, Type II NKT cells were sufficient to suppress tumor immunosurveillance in multiple mouse tumor models [50]. In this setting, Type II cells may be the primary driver of immune suppression. Similarly, in a murine model of obesity, transfer of Type II NKT cells into immunodeficient obese mice had a profoundly positive impact. Primarily, Type II cells induced a greater and more prolonged weight loss [51]. In addition, cell transfer resulted in a greater degree of glucose tolerance over the 6 day study interval. Type II cell activation has also been implicated in a transgenic model of hepatitis B as well as ulcerative colitis [52,53].

Cumulatively, little is known about the role of Type II NKT cells in liver inflammation and injury or the interactions with iNKT. Halder and colleagues reported that the activation of Type II cells resulted in the preferential activation of plasmacytoid dendritic cells, leading the Interleukin-12-mediated recruitment of iNKT cells into the mouse liver [54]. As a result, these recruited iNKT cells became anergized, in turn preventing the induction of Concanavalin-A-induced hepatitis. The authors point out that these data represent a unique cellular interaction leading to Type II-mediated regulation of iNKT cells. A similar regulatory role of Type II cells has been observed in tumor immunity [55].

A follow-up study by the same group employed a murine model of warm ischemia using an atraumatic clip technique [56]. After 90 min of partial warm ischemia, mice were euthanized following either 6 or 24 h of reperfusion. Type I NKT cells directly mediate hepatic IRI as mice lacking these cells are protected from injury in the setting of normal Type II NKT cell levels as early as 6 h post-reperfusion (reduction in serum ALT). At 24 h post-perfusion, NKT^−/−^ mice demonstrated dramatically reduced levels of tissue necrosis. Conversely, selective activation of Type II NKT cells directly prevents liver injury and is associated with the inhibition of IFN-γ secretion by iNKT cells as well as the suppression of myeloid cell recruitment to the liver. The authors suggest that this inhibition of myeloid recruitment may be linked to a reduction in hepatocyte and endothelial injury. However, it is unclear if this myeloid recruitment is dependent on specific cytokine secretion. These results above were summarized in Table 2.

## 6. Comment

In 2014, 14,632 patients with end-stage liver disease requiring transplant were on the UNOS waiting list in the United States [57]. Unfortunately, only 6729 liver transplants were performed in that same year. In the setting of a critical organ shortage, using marginal organs remains the predominant mechanism by which to expand the donor pool. Despite aggressive efforts to maximize the utilization of extended criteria organs, IRI leading to graft failure or dysfunction is still a major obstacle to transplantation. The temporal relationship in the axis of hypoxia-hepatocellular injury and innate/adaptive immune responses continues to be defined. At present, the specific cellular subsets involved in tissue injury are poorly understood. Furthermore, the specific mechanisms employed to induce direct cellular injury are just beginning to be uncovered. Exciting new observations center on the ability of NKT cells to directly recruit and activate NK cells to the liver. These observations are critical in that accumulating evidence suggests that NK cells can recruit and activate neutrophils as well as cause direct liver injury. In addition, the role of A_2A_R signaling has emerged as a potential mechanism by which post-perfusion inflammation can be influenced. Specifically, the role of adenosine receptor expression on NKT cells and subsequent cytokine production in the liver following reperfusion is virtually unknown. Cumulatively, the pro- versus anti-inflammatory influences of NKT cell subsets may provide several attractive therapeutic targets to promote hepatocellular protection in the setting of transplantation.

## Figures and Tables

**Table 1:** CD4+ T Lymphocytes and Warm Liver IRI.

Reference	Murine Models of IRI	Findings
[15]	30, 60 and 90 min partial ischemia. Reperfusion from 0–36 h.	Similar IRI in acute phase (3–6 h) btwn BALB/c and nu/nu mice. At 16–20 h enzymes/necrosis reduced in nu/nu (10-fold reduction in neutrophil accumulation). CD4^+^-depletion in BALB/c reduced IRI.
[16]	90 min partial ischemia. Reperfusion at 4 and 20 h.	IRI significantly reduced in T-cell deficiency, disruption of CD154 signaling, or CD154 blockade.
[17]	90 min partial ischemia. Reperfusion at 1, 4, and 8 h.	CD4^+^ lymphocytes were recruited to the liver within 1 h of reperfusion. CD4^−/−^ had greater injury and less neutrophil accumulation.
[18]	90 min partial ischemia. Reperfusion at 30–140 min.	CD4 deficiency, CD40-CD40L and CD28-B7 disruption attenuates platelet adherence, reduces neutrophil transmigration, sinusoidal perfusion failure, and transaminase activities.
[19]	90 min partial ischemia. Reperfusion at 8 h.	CD4^−/−^ and CD4 depletion protect from IRI. CD154 blockade protects from IRI. CD4 T cells function in IRI without de novo Ag-specific activation.
[20]	60 min partial ischemia. Reperfusion 0–24 h.	Anti-CD25 mAb protects from IRI via reducing CD4^+^ T cells (less expression of TNF-∝, IFN-γ, IL-2, and IL-6).

IFN-γ = interferon gamma; TNF-α = tumor necrosis factor-α; IL-2 = interleukin-2; IL-6 = interleukin-6; IRI = ischemia reperfusion injury.

**Table 2:** NKT cells in warm hepatic IRI.

Reference	Murine Models of IRI	Findings
[37]	30 min portal vein clamping, reperfusion times up to 50 h.	Reduction in liver injury (sALT) by 50% in NKT^−/−^ mice. Serum IFN-γ reduced in NKT^-/-^ mice.
[38]	72 min partial ischemia, reperfusion at 2, 24, and 48 h.	NKTc produce IFN-γ at 2 h. NK/NKTc depletion reduces sALT at 24 h. Adoptive transfer of NKTc from WT or A_2A_R KO restores IRI in RAG-1 mice.
[40]	90 min partial ischemia, reperfusion at 4 and 8 h.	NKTc (not NK) contribute to hepatic IRI by an anti-CD1d-dependent activationo of TCRs. NKTc depletion attenuated IRI.
[45]	60 min partial ischemia, 6 h reperfusion.	Selective activation of NKTc 1 h prior to ischemia reduced IRI. Protection is via an IL-13/A_2A_R-dependent mechanism.
*Type II NKTc* [56]	90 min partial ischemia. Reperfusion at 6 and 24 h.	Mice lacking Type I NKTc were protected from IRI. Type II NKTc activation reduced IFN-γ secretion by Type I NKTc and prevented IRI.

NKTc = natural killer T cell; IFN-γ = interferon gamma; sALT = serum alanine aminotransferase; TCR = T cell receptor; IRI = ischemia reperfusion injury; A2AR = adenosine 2A receptor.

## References

[1] Zimmerman M.A., Kam I., Eltzschig H., Grenz A. (2013). Biological implications of extracellular adenosine in hepatic ischemia and reperfusion injury. Am. J. Transplant..

[2] Zhai Y., Petrowsky H., Hong J.C., Busuttil R.W., Kupiec-Weglinski J.W. (2013). Ischaemia-reperfusion injury in liver transplantation—From bench to bedside. Nat. Rev. Gastroenterol. Hepatol..

[3] Datta G., Fuller B.J., Davidson B.R. (2013). Molecular mechanisms of liver ischemia reperfusion injury: Insights from transgenic knockout models. World J. Gastroenterol..

[4] Zhai Y., Busuttil R.W., Kupiec-Weglinski J.W. (2011). Liver ischemia and reperfusion injury: New insights into mechanisms of innate-adaptive immune-mediated tissue inflammation. Am. J. Transplant..

[5] Hernandez L.A., Grisham M.B., Twohig B., Arfors K.E., Harlan J.M., Granger D.N. (1987). Role of neutrophils in ischemia-reperfusion-induced microvascular injury. Am. J. Physiol..

[6] Jaeschke H., Farhood A., Smith C.W. (1990). Neutrophils contribute to ischemia/reperfusion injury in rat liver in vivo. FASEB J..

[7] Komatsu H., Koo A., Ghadishah E., Zeng H., Kuhlenkamp J.F., Inoue M., Guth P.H., Kaplowitz N. (1992). Neutrophil accumulation in ischemic reperfused rat liver: Evidence for a role for superoxide free radicals. Am. J. Physiol..

[8] Langdale L.A., Flaherty L.C., Liggitt H.D., Harlan J.M., Rice C.L., Winn R.K. (1993). Neutrophils contribute to hepatic ischemia-reperfusion injury by a CD18-independent mechanism. J. Leukoc. Biol..

[9] Suzuki S., Toledo-Pereyra L.H., Rodriguez F.J. (1994). Role of neutrophils during the first 24 h after liver ischemia and reperfusion injury. Transplant. Proc..

[10] Jaeschke H., Bautista A.P., Spolarics Z., Spitzer J.J. (1991). Superoxide generation by Kupffer cells and priming of neutrophils during reperfusion after hepatic ischemia. Free Radic. Res. Commun..

[11] Jaeschke H., Farhood A., Bautista A.P., Spolarics Z., Spitzer J.J., Smith C.W. (1993). Functional inactivation of neutrophils with a Mac-1 (CD11b/CD18) monoclonal antibody protects against ischemia-reperfusion injury in rat liver. Hepatology.

[12] Doherty D.G., O’Farrelly C. (2000). Innate and adaptive lymphoid cells in the human liver. Immunol. Rev..

[13] Norris S., Collins C., Doherty D.G., Smith F., McEnte G., Traynor O., Nolan N., Hegarty J., O’Farrelly C. (1998). Resident human hepatic lymphocytes are phenotypically different from circulating lymphocytes. J. Hepatol..

[14] Caldwell C.C., Tschoep J., Lentsch A.B. (2007). Lymphocyte function during hepatic ischemia/reperfusion injury. J. Leukoc. Biol..

[15] Zwacka R.M., Zhang Y., Halldorson J., Schlossberg H., Dudus L., Engelhardt J.F. (1997). CD4(+) T-lymphocytes mediate ischemia/reperfusion-induced inflammatory responses in mouse liver. J. Clin. Investig..

[16] Shen X.D., Ke B., Zhai Y., Amersi F., Gao F., Anselmo D.M., Anselmo D.M., Busuttil R.W., Kupiec-Weglinski J.W. (2002). CD154-CD40 T-cell costimulation pathway is required in the mechanism of hepatic ischemia/reperfusion injury, and its blockade facilitates and depends on heme oxygenase-1 mediated cytoprotection. Transplantation.

[17] Caldwell C.C., Okaya T., Martignoni A., Husted T., Schuster R., Lentsch A.B. (2005). Divergent functions of CD4^+^ T lymphocytes in acute liver inflammation and injury after ischemia-reperfusion. Am. J. Physiol. Gastrointest. Liver Physiol..

[18] Khandoga A., Hanschen M., Kessler J.S., Krombach F. (2006). CD4^+^ T cells contribute to postischemic liver injury in mice by interacting with sinusoidal endothelium and platelets. Hepatology.

[19] Shen X., Reng F., Gao F., Uchida Y., Busuttil R.W., Kupiec-Weglinski J.W., Zhai Y. (2010). Alloimmune activation enhances innate tissue inflammation/injury in a mouse model of liver ischemia/reperfusion injury. Am. J. Transplant..

[20] Yang J., Wang X., Song S., Liu F., Fu Z., Wang Q. (2014). Near-term anti-CD25 monoclonal antibody administration protects murine liver from ischemia-reperfusion injury due to reduced numbers of CD4^+^ T cells. PLoS ONE.

[21] Viale R., Ware R., Maricic I., Chaturvedi V., Kumar V. (2012). NKT Cell Subsets Can Exert Opposing Effects in Autoimmunity, Tumor Surveillance and Inflammation. Curr. Immunol. Rev..

[22] Li L., Huang L., Sung S.S., Lobo P.I., Brown M.G., Gregg R.K., Engelhard V.H., Okusa M.D. (2007). NKT cell activation mediates neutrophil IFN-gamma production and renal ischemia-reperfusion injury. J. Immunol..

[23] Sharma A.K., LaPar D.J., Zhao Y., Li L., Lau C.L., Kron I.L., Iwakura Y., Okusa M.D., Laubach V.E. (2011). Natural killer T cell-derived IL-17 mediates lung ischemia-reperfusion injury. Am. J. Respir. Crit. Care Med..

[24] Kita H., Naidenko O.V., Kronenberg M., Ansari A.A., Rogers P., He X.S., Koning F., Mikayama T., Van de Water J., Coppel R.L. (2002). Quantitation and phenotypic analysis of natural killer T cells in primary biliary cirrhosis using a human CD1d tetramer. Gastroenterology.

[25] Tsuneyama K., Yasoshima M., Harada K., Hiramatsu K., Gershwin M.E., Nakanuma Y. (1998). Increased CD1d expression on small bile duct epithelium and epithelioid granuloma in livers in primary biliary cirrhosis. Hepatology.

[26] Chuang Y.H., Lian Z.X., Yang G.X., Shu S.A., Moritoki Y., Ridgway W.M., Ansari A.A., Kronenberg M., Flavell R.A., Gao B. (2008). Natural killer T cells exacerbate liver injury in a transforming growth factor beta receptor II dominant-negative mouse model of primary biliary cirrhosis. Hepatology.

[27] Wu S.J., Yang Y.H., Tsuneyama K., Leung P.S.C., Illarionov P., Gershwin M.E., Chuang Y.H. (2011). Innate immunity and primary biliary cirrhosis: activated invariant natural killer T cells exacerbate murine autoimmune cholangitis and fibrosis. Hepatology.

[28] Ishikawa S., Ikejima K., Yamagata H., Tomonori A., Kon K., Arai K., Takeda K., Watanabe S. (2011). CD1d-restricted natural killer T cells contribute to hepatic inflammation and fibrogenesis in mice. J. Hepatol..

[29] Syn W.K., Agboola K.M., Swiderska M., Michelotti G.A., Liaskou E., Pang H., Xie G., Philips G., Chan I.S., Karaca G.F. (2012). NKT-associated hedgehog and osteopontin drive fibrogenesis in non-alcoholic fatty liver disease. Gut.

[30] Kumar V. (2013). NKT-cell subsets: Promoters and protectors in inflammatory liver disease. J. Hepatol..

[31] Swain M.G. (2008). Hepatic NKT cells: Friend or foe?. Clin. Sci..

[32] Eberl G., Lees R., Smiley S.T., Taniguchi M., Grusby M.J., MacDonald H.R. (1999). Tissue-specific segregation of CD1d-dependent and CD1d-independent NK T cells. J. Immunol..

[33] Klugewitz K., Adams D.H., Emoto M., Eulenburg K., Hamann A. (2004). The composition of intrahepatic lymphocytes: Shaped by selective recruitment?. Trends Immunol..

[34] Van Kaer L. (2007). NKT cells: T lymphocytes with innate effector functions. Curr. Opin. Immunol..

[35] Bendelac A., Savage P.B., Teyton L. (2007). The biology of NKT cells. Ann. Rev. Immunol..

[36] Kronenberg M., Gapin L. (2002). The unconventional lifestyle of NKT cells. Nat. Rev. Immunol..

[37] Shimamura K., Kawamura H., Nagura T., Kato T., Naito T., Kameyama H., Hatakeyama K., Abo T. (2005). Association of NKT cells and granulocytes with liver injury after reperfusion of the portal vein. Cell. Immunol..

[38] Lappas C.M., Day Y.J., Marshall M.A., Engelhard V.H., Linden J. (2006). Adenosine A_2A_ receptor activation reduces hepatic ischemia reperfusion injury by inhibiting CD1d-dependent NKT cell activation. J. Exp. Med..

[39] Day Y.J., Marshall M.A., Huang L., McDuffie M.J., Okusa M.D., Linden J. (2004). Protection from ischemic liver injury by activation of A_2A_ adenosine receptors during reperfusion: Inhibition of chemokine induction. Am. J. Physiol. Gastrointest. Liver Physiol..

[40] Kuboki S., Sakai N., Tschop J., Edwards M.J., Lentsch A.B., Caldwell C.C. (2009). Distinct contributions of CD4^+^ T cell subsets in hepatic ischemia/reperfusion injury. Am. J. Physiol. Gastrointest. Liver Physiol..

[41] Carini R., Albano E. (2003). Recent insights on the mechanisms of liver preconditioning. Gastroenterology.

[42] Koti R.S., Seifalian A.M., Davidson B.R. (2003). Protection of the liver by ischemic preconditioning: A review of mechanisms and clinical applications. Dig. Surg..

[43] Pasupathy S., Homer-Vanniasinkam S. (2005). Surgical implications of ischemic preconditioning. Arch. Surg..

[44] Selzner N., Rudiger H., Graf R., Clavien P.A. (2003). Protective strategies against ischemic injury of the liver. Gastroenterology.

[45] Cao Z., Yuan Y., Jeyabalan G., Du Q., Tsung A., Geller D.A., Billiar T.R. (2009). Preactivation of NKT cells with alpha-GalCer protects against hepatic ischemia-reperfusion injury in mouse by a mechanism involving IL-13 and adenosine A_2A_ receptor. Am. J. Physiol. Gastrointest. Liver Physiol..

[46] Emoto M., Kaufmann S.H. (2003). Liver NKT cells: An account of heterogeneity. Trends Immunol..

[47] Godfrey D.I., MacDonald H.R., Kronenberg M., Smyth M.J., Van Kaer L. (2004). NKT cells: What’s in a name?. Nat. Rev. Immunol..

[48] Jahng A., Maricic I., Aguilera C., Cardell S., Halder R.C., Kumar V. (2004). Prevention of autoimmunity by targeting a distinct, noninvariant CD1d-reactive T cell population reactive to sulfatide. J. Exp. Med..

[49] Bandyopadhyay K., Marrero I., Kumar V. (2016). NKT cell subsets as key participants in liver physiology and pathology. Cell. Mol. Immunol..

[50] Terabe M., Swann J., Ambrosino E., Sinha P., Takaku S., Hayakawa Y., Godfrey D.I., Ostrand-Rosenberg S., Smyth M.J., Berzofsky J.A. (2005). A nonclassical non-Valpha14Jalpha18 CD1d-restricted (type II) NKT cell is sufficient for down-regulation of tumor immunosurveillance. J. Exp. Med..

[51] Hams E., Locksley R.M., McKenzie A.N., Fallon P.G. (2013). Cutting edge: IL-25 elicits innate lymphoid type 2 and type II NKT cells that regulate obesity in mice. J. Immunol..

[52] Baron J.L., Gardiner L., Nishimura S., Shinkai K., Locksley R., Ganem D. (2002). Activation of a nonclassical NKT cell subset in a transgenic mouse model of hepatitis B virus infection. Immunity.

[53] Fuss I.J., Heller F., Boirivant M., Leon F., Yoshida M., Fichtner-Feigl S., Yang Z., Exley M., Kitani A., Blumberg R.S. (2004). Nonclassical CD1d-restricted NK T cells that produce IL-13 characterize an atypical Th2 response in ulcerative colitis. J. Clin. Investig..

[54] Halder R.C., Aguilera C., Maricic I., Kumar V. (2007). Type II NKT cell-mediated anergy induction in type I NKT cells prevents inflammatory liver disease. J. Clin. Investig..

[55] Ambrosino E., Terabe M., Halder R.C., Peng J., Shun T., Miyake S., Yamaura T., Kumar V., Berzofsky J.A. (2007). Cross-regulation between type I and type II NKT cells in regulating tumor immunity: A new immunoregulatory axis. J. Immunol..

[56] Arrenberg P., Maricic I., Kumar V. (2011). Sulfatide-mediated activation of type II natural killer T cells prevents hepatic ischemic reperfusion injury in mice. Gastroenterology.

[57] Kim W.R., Lake J.R., Smith J.M., Skeans M.A., Schladt D.P., Edwards E.B., Harper A.M., Wainright J.L., Snyder J.J., Israni A.K. (2016). Liver. Am. J. Transplant..

